# Design and Development of Magnetic Iron Core Gold Nanoparticle-Based Fluorescent Multiplex Assay to Detect *Salmonella*

**DOI:** 10.3390/nano12213917

**Published:** 2022-11-07

**Authors:** Xinyi Zhao, Gwendoline Smith, Bilal Javed, Garret Dee, Yurii K. Gun’ko, James Curtin, Hugh J. Byrne, Christine O’Connor, Furong Tian

**Affiliations:** 1 School of Food Science & Environmental Health, Technological University Dublin, Grangegorman, D07 H6K8 Dublin, Ireland; 2 FOCAS Research Institute, Technological University Dublin, Camden Row, D08 CKP1 Dublin, Ireland; 3 AMBER, Trinity College Dublin, D02 PN40 Dublin, Ireland; 4 Faculty of Engineering and Built Environment, Technological University Dublin, Bolton Street, D01 K822 Dublin, Ireland

**Keywords:** bacterial incubation, protein conjugation, iron oxide nanoparticle (IONP), iron core gold nanoparticle (ICGNP), *Salmonella*, food contaminants

## Abstract

*Salmonella* is a bacterial pathogen which is one of the leading causes of severe illnesses in humans. The current study involved the design and development of two methods, respectively using iron oxide nanoparticle (IONP) and iron core gold nanoparticle (ICGNP), conjugated with the Salmonella antibody and the fluorophore, 4-Methylumbelliferyl Caprylate (4-MUCAP), used as an indicator, for its selective and sensitive detection in contaminated food products. Twenty double-blind beverage samples, spiked with *Salmonella enteritidis*, *Staphylococcus aureus*, and *Escherichia coli*, were prepared in sterile Eppendorf^®^ tubes at room temperature. The gold layer and spikes of ICGNPs increased the surface areas. The ratio of the surface area is 0.76 (IONPs/ICGNPs). The comparative sensitivity and specificity of the IONP-based and the ICGNP-based methods to detect *Salmonella* were determined. The ICGNP method shows the limit of detection is 32 *Salmonella* per mL. The ICGNPs had an 83.3% sensitivity and a 92.9% specificity value for the presence and detection of *Salmonella*. The IONP method resulted in a limit of detection of 150 *Salmonella* per mL, and a 66.7% sensitivity and 83.3% specificity for the presence and detection of *Salmonella*. The higher surface area of ICGNPs increases the efficiency of detection. The monitoring of *Salmonella* can thus be achieved by a rapid magnetic fluorescent assay using a smartphone for image capture and analyze, providing quantitative results. The findings from the present study would help to detect *Salmonella* rapidly in water. It can improve the microbial quality of water and food safety due to the presence of *Salmonella* in the water environment.

## 1. Introduction

In Europe, almost 23 million people fall sick and around 4700 people die due to consumption of contaminated food every year. *Salmonella* is reported to be the most severe pathogen, causing death in most cases. *Salmonella* is a pathogen, infection with which can result in weakness, vomiting, dehydration, etc. in humans and other animals and it has the capacity to damage various cells of the body e.g., macrophages, hepatocytes, and neutrophils [1]. Humans and domestic animals come in contact with *Salmonella* mainly from grain or feeders, irrigation water, vehicles for transport, and surface waters contaminated by animals and birds [2]. *Salmonella* can persist in rivers, lakes, ponds, and well waters, particularly in a rural areas in developing countries [3]. There are many strains of *Salmonella*, the most common of which is *Salmonella enteritidis* [4]. *Staphylococcus aureus* and *Escherichia coli* are also very common bacterial pathogens and cause mild symptoms [5,6].

The traditional method for the detection of *Salmonella* is bacterial culture, which entails isolating the bacteria and monitoring the growth of the colonies, a procedure which is very time-consuming [7]. In order to identify different types of bacterial contamination during culture, the bacterial colonies are fixed and stained on a glass slide and confirmed by microscopy observation. The Enzyme-linked immunosorbent assay (ELISA), available as a commercial test kit, is currently the most used method for the detection of the presence of *Salmonella*. It has drawbacks, however, such as low sensitivity [8]. Polymerase chain reaction (PCR) is more sensitive, with a detection limit as low as 1000 CFU/mL. However, it is time-consuming, expensive, labor-intensive, and needs highly qualified staff to operate the laboratory equipment [9].

The Lateral Flow Immunochromatographic Assay (LFIA) is a relatively new method for the detection of *Salmonella* and is very rapid. It requires only 4 to 5 min to complete the detection process and has a high specificity (e.g., 50,000 CFU/mL) [10]. LFIA is cost-effective, simple to use, and can produce results rapidly with fewer samples [11,12]. The conjugated particles, as an indicator, flow through the porous membrane [13,14]. Gold nanoparticles (GNPs) are the most used NPs in the LFIA because of their high stability, easily modified microstructure, and distinct color [10]. GNPs have very low toxicity, and their particle size can be controlled by various factors such as the temperature, pH, ratio, concentration of reactants, and mechanical forces [15]. On the other hand, one of the most used NPs in the microbial quality of water and food safety area is the Iron Oxide NP (IONP), which has superparamagnetic properties, and is in abundance since iron is one of the core elements of the earth [16]. IONPs are frequently used in environmental science because of their low toxicity, good biocompatibility, and they can be easily modified by bioactive molecules such as proteins, antibodies, and nucleic acids, etc. [17]. Moreover, their superparamagnetic nature makes them a suitable candidate to be used in medicine and diagnostics [18].

Theoretically, the combination of IONP and GNP can be achieved, which would result in the synthesis of highly functionalized NPs with enhanced optical and catalytic properties [19]. Using gold as a coating on IONP has benefits such as lower nonspecific protein adsorption, higher specificity, etc., and will affect the functionality of the magnetism [20].

This study involves the design and development of a novel method of using IONP and ICGNP to detect the presence of *S. enteritidis* in water-based beverages. Firstly, the IONPs have been synthesized by the reduction method, while the gold shell will be added by the seed growth method [21]. Both IONPs and the ICGNPs have been conjugated with the Anti-Salmonella antibody and bacteria, after which characterization of these NPs and bacteria was performed to determine their morphological and optical characteristics. The fluorophore, 4-Methylumbelliferyl Caprylate (4-MUCAP) was employed as an indicator of the presence of *Salmonella* [22]. *S. aureus* and *E. coli* were used as negative control samples, to test the specificity of the detection.

## 2. Materials and Methods

### 2.1. Chemicals and Materials

The chemicals used in the study were of analytical grade without any further modification. GNPs (diameter: 10 nm, concentration: 6 × 10^12^/mL, solvent: 0.1 mM phosphate buffered saline (PBS), 1-ethyl-3-(3-dimethylaminopropyl) carbodiimide hydrochloride (EDC), tetrachloroauric acid trihydrate (HAuCl_4_), N-Hydroxy succinimide (NHS), dimethyl sulfoxide (DMSO), ammonium chloride (NH_4_Cl), 25% of ammonium solution, iron dichloride (FeCl_2_), iron trichloride (FeCl_3_), sodium citrate, hydroquinone, trisodium citrate were purchased from Sigma-Aldrich. *S. enteritidis* (ATCC13076), *S. aureus* (ATCC 25923), and *E. coli* (ATCC 25922) were cultured. The fluorophore, 4-methylumbelliferyl caprylate (4-MUCAP) was obtained from LGC Ltd., Middlesex, UK).

### 2.2. Synthesis of IONPs and ICGNPs

Firstly, the IONPs were synthesized. 30 g of NH_4_Cl was dissolved in 150 mL of ultrapure water and further degassed under argon. 40 mL of 25% ammonia solution was added to the solution. This solution was placed on a heating mantel at 90 °C with mechanical stirring under argon. A quantity of 0.86 g of FeCl_2_ and 2.33 g of FeCl_3_ were added to the 25 mL of degassed ultrapure water and sonicated under argon until they were dissolved. The iron solution was then added dropwise to the ammonia solution under argon and heat while being stirred continuously. The mixed solution was further heated for 2 h at 90 °C under argon. The product underwent magnetic separation where it was washed with degassed ultrapure water and methanol. The IONPs were coated with citric acid by dispersing the nanoparticles in a 0.1 g/mL citric acid solution. The reaction mixture was stirred for 3 h at a temperature of 80 °C. The solution was then cooled, the IONP–COOH were precipitated using a magnet, washed with deionized water, and dried. The IONP–COOH were stored at 2.5 mg/mL in ultrapure water for further use.

The synthesis of the ICGNPs was carried out following the seed growth method [21]. 10 μL of the 2.5 mg/mL IONPs–COOH in ultrapure water solution were further diluted in 0.5 mL of ultrapure water. This corresponds to 9.26 × 1012 particles used as seeds. The 0.5 mL diluted IONPs–COOH, 400 μL of 10 mmol/L HAuCl4, 0.2 mL of 800 μmol/L sodium citrate solution, and 1.5 mL of 400 mmol/L hydroquinone solution were mixed to a total volume of 200 mL in water and allowed to react at room temperature for 30 min without stirring. The ICGNPs product underwent magnetic separation, was washed with deionized water, dried, and weighed. 5.4 mg of ICGNP were obtained. The ICGNPs were coated with citric acid by dispersing the nanoparticles in a 0.1 g/mL citric acid solution by sonication. The reaction mixture was stirred for 3 h at a temperature of 80 °C. The solution was then cooled, and the magnet was placed on the outside of the glassware to precipitate ICGNP–COOH. ICGNP–COOH were washed with deionized water for further use.

### 2.3. Conjugation Carbodiimide

**Figure 1:**
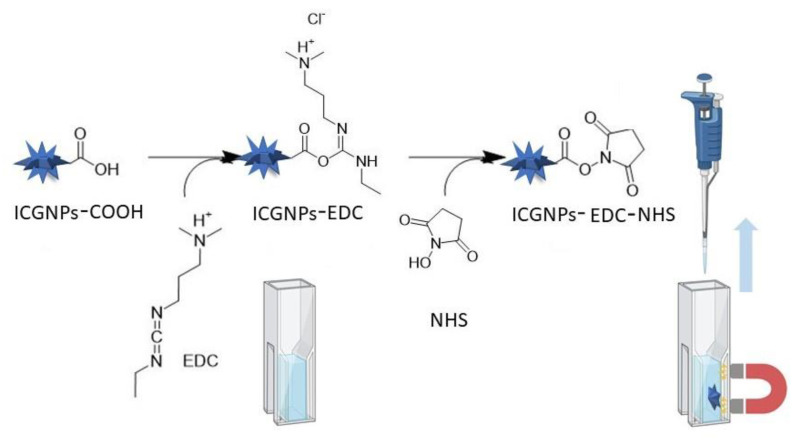
Schematic representation of conjugation of ICGNPs in a cuvette. NHS and EDC can react with ICGNP–COOH to form IGGNP–EDC-NHS. IGGNP–EDC-NHS can then be removed from the suspension by a magnet applied to the external surface. IGGNP–EDC-NHS provides the amide to connect with antibody.

The anti-Salmonella antibody conjugation is performed by following the carbodiimide conjugation technique [23]. The IONP–COOH, and ICGNP–COOH were obtained from Section 2.2. Figure 1 depicts the conjugation of EDC, and NHS with ICGNPs. O-acylisourea can react with ICGNP-COOH to give the amide to connect with the antibody. A quantity of 2 mg of ICGNP–COOH (3.7 × 1012 particles) was added to the 4 mL of the cuvette, then 20 mg of NHS and EDC were added to the same cuvette. A magnet was applied to the outside of the cuvette to attract the IGGNP–EDC-NHS. This was followed by the addition of 1 mL of ultrapure water which had been autoclaved. This was left in the incubator for 1 h at 37 °C for magnetic separation. The magnet was placed on the outside of the cuvette which helped the IGGNP–EDC-NHS to adhere to it. Then, the IGGNP–EDC-NHS were rinsed with ultrapure water 3 times to wash the particles. The 20 g anti-Salmonella antibody (AntiB) with 1 mL buffer (pH 7) was added to the same cuvette and left to incubate for 1 h at 37 °C. The magnet was once again placed on the outside of the cuvette, the solution was decanted, and the ICGNP– EDC-NHS-AntiB were washed with ultrapure water, followed by pH 9 buffer solution and then pH 7 buffer. After all washes, the ICGNP–EDC-NHS-AntiB were left in ultrapure water for further use. 20 μL of IONP–COOH from Section 2.2 were extracted and underwent the same conjugation procedure. The number of particles used in the reaction was 1.85 × 10^13^, 5 times the number for ICGNPs, yielding a light brown color suspension.

### 2.4. Optical and Physical Characteristics of Nanomaterials

Particle sizes of the NPs were determined by a Zetasizer Nano ZS analyzer (Malvern Instruments, Worcestershire, UK). The samples were prepared in deionized water and disposable cuvettes were used for measurement. Transmission Electron Microscope (TEM) images were obtained by a JEOL 2100 Lab TEM (JEOL Ltd., Tokyo, Japan) operating at 200 kV with a LaB6 electron source. The Vibrating Sample Magnetometer (VSM) graph was obtained by an in-house assembled VSM with a maximum field of 1.1 T from the Amber Centre in Trinity College Dublin. The X-ray powder diffraction (XRD) analysis was obtained by a Bruker, D2 Phaser 2nd generation, pXRD (Bruker Ltd., Billerica, MA, USA) with a copper X-ray source of wavelength 0.154 nm. Carboxylation of the NP surfaces was confirmed by Attenuated Total Reflection Fourier Transform infrared spectroscopy (ART-FTIR) (Thermo Fisher Scientific, Cork, Ireland). Aqueous suspensions of ICGNP and ICGNP–COOH were concentrated with a magnet as measured as drops on the ATR crystal. Distilled water was used as a reference.

### 2.5. Bacterial Sample Preparation

*S. enteritidis* (ATCC13076), *S. aureus* (ATCC 25923), and *E. coli* (ATCC 25922) were cultured by initially spreading the bacteria on tryptic soy agar (TSA, Scharlau Chemie) and incubating them overnight at 37 °C. Later, the bacterial cells were harvested in sterile ultrapure water and centrifuged at 8720× *g* for 10 min. The cell pellet was washed twice with sterile ultrapure water and resuspended at a concentration of 1 to 100 CFU/mL. The bacterial density was determined by measuring the absorbance at 550 nm using the McFarland standard (BioMerieux, Marcy-l’Etoile, France). The three bacterial strains were subjected to the IONP and ICGNP detection methods. 3 different concentrations (10^−4^ to 10^−6^ units) of each of the 3 different bacteria were prepared. The bacteria are captured when exposed to the IONP–EDC-NHS-AntiB or ICGNP–EDC-NHS-AntiB and can therefore be isolated and removed from the solution.

### 2.6. Layout of Bacterial Concentrations in Cuvette

The limits of detection of bacteria by IONPs or ICGNPs were determined according to colony number counts. First, the colony growth without nanoparticles (NPs) was counted, to indicate the total number of bacteria. The colony growth with IONPs or ICGNPs was analyzed after bacteria solution enrichment with magnets in the cuvettes. The detail of the sample layout was described. There was a total of 54 Eppendorf^®^ tubes to analyze, containing blank without NPs, with IONPs, and ICGNPs, for the 6 different concentrations (10^−1^ to 10^−6^ units) of the 3 different bacteria. 100 μL of conjugated IONPs or ICGNPs were added into 18 different cuvettes, respectively. The Eppendorf^®^ tubes of the first 18 were without nanoparticles, containing 100 µL of ultrapure water. The three bacteria *S. enteritidis*, *S. aureus*, and *E. coli* were diluted from 10^−1^ to 10^−6^ units. 0.9 mL of the diluted bacterial solution was added to the cuvette. The magnets were employed to enrich the bacteria. The magnet was applied to each sample with or without nanoparticles (1 mL) for 10 min to enrich the bacteria, attached to NPs. Then, 0.9 mL of cleared solution was removed by pipette. The 0.9 mL of water was added to the enriched sample (0.1 mL) to make a total of 1 mL. The sample was suspended in the cuvette. Then, 0.1 mL of 1 mL bacterial solution was spread on an agar plate for 24 h incubation. In parallel, the 0.1 mL of 1 mL total bacterial solution was added with fluorescent dye in Section 2.7 visibility study with the fluorescent dye. Three replicates were measured for each sample.

### 2.7. Visibility with Fluorescent Dye

100 mM 4-MUCAP solutions were prepared in 1 mL of DMSO (99.5%) and kept at 4 °C. Further dilutions were prepared in ultrapure water with a final DMSO concentration of 1%. Different diluted bacterial solutions, after enrichment according to Section 2.6, were injected into the cuvette containing 0.9 mL of buffered 4-MUCAP substrate solution (pH 6.8). The magnets were employed to enrich the 4-MUCAP. The magnet was applied to each sample (1 mL) for 10 min to enrich the dye. Then, 0.9 mL of cleared solution was removed by pipette. The 0.9 mL of water were added into the dye enriched sample (0.1 mL) to make a total of 1 mL. The sample was suspended in the cuvette. Images were then taken by using a smartphone in light and dark conditions.

### 2.8. Sensitivity and Specificity Test

20 double-blind samples with *Salmonella* and without *Salmonella* solution were prepared in sterile Eppendorf^®^ tubes at room temperature. The 4-MUCAP fluorogenic substrate presents blue fluorescence when exposed to *Salmonella*, but not *S. aureus*, or *E. coli* [24]. Two-tail analysis with Fisher’s test was employed to evaluate the prediction given, indicating that the method can successfully predict the outcome. The bacteria agar plate culture was employed as the gold standard to indicate true positive and true negative values. The sensitivity and specificity of the IONP method and the ICGNP method in Section 2.7 for *Salmonella* detection were compared. For the Specificity Test, experiments were also done on *S. aureus* and *E. coli*.

## 3. Results

### 3.1. Optical and Morphological Characterizations of NPs

**Figure 2:**
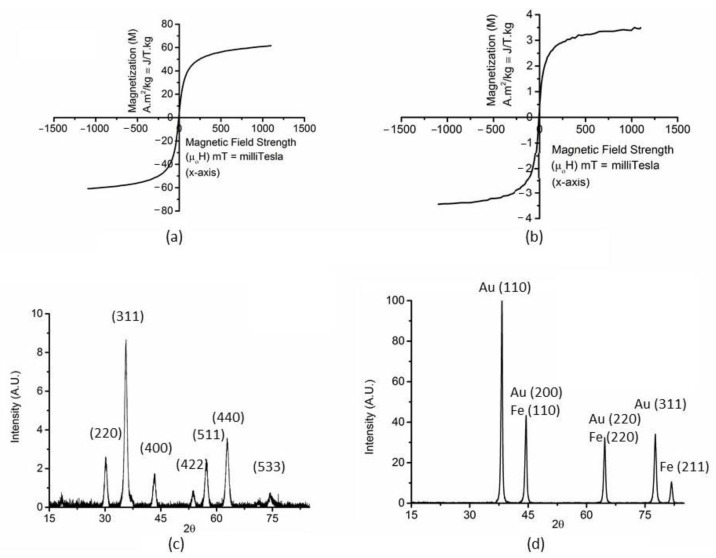
Characterizations of IONPs and ICGNPs: (**a**) VSM, IONPs. (**b**) VSM, ICGNPs. (**c**) XRD, IONPs. (**d**) XRD, ICGNPs.

The optical and morphological characterization of nanomaterials was performed by using various material characterization techniques. VSM was conducted to determine the magnetization of IONPs and ICGNPs (Figure 2a,b). The magnetizations were found to be 61 Am^2^/kg for IONP and 3.5 Am^2^/kg for ICGNP at 1 A/m. The hysteresis loop had no remnant magnetization for either IONPs or ICGNPs at 0 A/m, indicating that both IONP and ICGNP were superparamagnetic. In order to confirm the core and shell structure, the XRD of IONPs and ICGNPs was investigated. There were many peaks at 2θ = ~30°, ~35°, ~43°, ~53°, ~57°, ~63°, ~74° in XRD of IONPs. Those peaks of crystalline iron correspond to Fe (220), (311), (400), (422), (511), (440), (533) in IONPs from both fcc and bcc characteristics (Figure 2c). However, XRD pattern of ICGNPs exhibited peaks at 2θ = ~38°, ~45°, ~65 °, ~78° and ~82°. The peaks at 2θ = ~38°, ~45°, ~65 °and ~78° were characteristic of gold crystalline diffraction, corresponding to Au (111), (200), (220), and (311) (Figure 2d).

The Bragg reflections indicated the formation of ICGNPs with a face-centered-cubic (fcc) lattice structure [25,26,27,28]. There were peaks hidden under the pattern of gold due to the overlap of their diffraction peaks at 45°, 65°, and 82°. The peak at 45°, 65°and 82°was indicated Fe (110), (220), and (211) [29]. The XRD confirmed ICGNP with an iron core and a full layer of gold shell.

**Figure 3:**
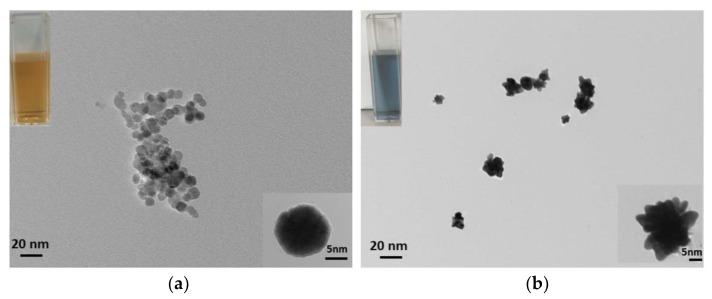
TEM images of (**a**) IONP and (**b**) ICGNP. The insert images on the left top corner illustrate the color of NPs in ultrapure water after synthesis. The insert images at the bottom corner are high magnificent images of individual nanoparticles.

TEM images illustrated the structure and shape of the NPs. In Figure 3a, IONP presented a spherical shape with a diameter of ~10 nm. In Figure 3b, ICGNP is presented as individual flower/spikes shaped particles with dimensions of ~30 nm (right bottom of insert image). The insert images show that, in the cuvettes, the IONP were yellow and ICGNP were blue in solution.

The large images with scale bars were shown in Figure S1. In order to study the particle geometry, ImageJ was employed. The particle area, length and angle of spike were determined and collated (see Figure S1). ICGNP typically have a central core and 15 vertices in a 3-dimensional arrangement. The angle at the vertex was 41°. The length of the vertices is averaged at 7 nm from (Table S1).

Based on idealised geometric structures, the calculation of the surface area of ICGNP with cone shaped spikes is illustrated schematically in Figure S2. The IONPs, shown as red spherical nanoparticles of diameter 22 nm, act as seeds for the further growth of ICGNPs. To calculate the surface area of the ICGNPs, the core was assumed to have a perfect spherical geometry. Each of the N spikes on the surface of the core was calculated as a cone, having a height of 7 nm and a base of radius 2.5 nm. The base was excluded from the total surface area. The number of spikes were counted by Image J (Figure S1 and Table S1). The average total surface area of an ICGNP was calculated to be 2059 nm^2^.

**Figure 4:**
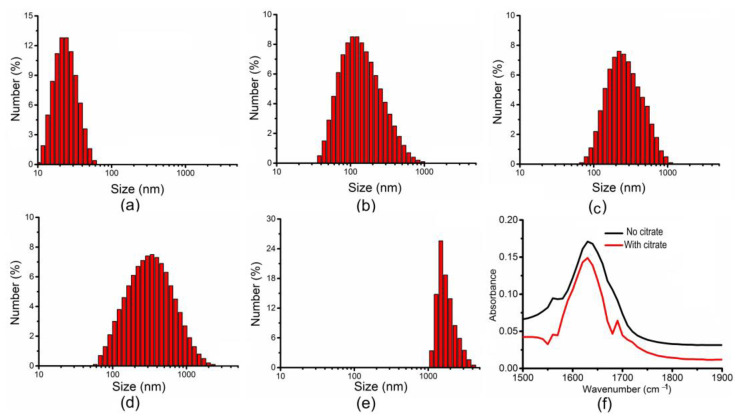
Particle size distribution and FTIR of ICGNPs (**a**) ICGNPs; (**b**) ICGNP–COOH; (**c**) ICGNP–EDC-NHS; (**d**) ICGNP–EDC-NHS-AntiB; (**e**) ICGNP–EDC-NHS-AntiB–Bacteria. (**f**) FTIR absorbance. The ICGNP–COOH with citrate in red curve. The ICGNP without citrate is in the black curve.

Figure 4a–e shows the particle size distribution of ICGNPs under different conditions. The centre of the distribution of ICGNP–COOH was increased to 100 nm after conjugation with -COOH. The centre of distribution of ICGNP–EDC-NHS was increased to 185 nm after conjugation with EDC and NHS. The centre of distribution of ICGNP–EDC-NHS-AntiB was increased to 300 nm after conjugation with antibody. When ICGNP was conjugated with *Salmonella* and its antibody, the centre of the distribution of ICGNP–EDC-NHS-AntiB–Bact was further increased to 1500 nm. The ICGNP and ICGNP–COOH were concentrated with a magnet from a water solution for FTIR analysis. Although the FTIR spectrum in both cases is dominated by the broad water absorption feature at ~1640 cm^−1^, that of ICGNP after treatment with citrate (red curve) shows a clear peak at 1700 cm^−1^, with absorbance of 0.065 compared to that of the ICGNP without citrate(black curve) (Figure 4f), confirming the presence of the carboxylic functional groups which can serve as sites to initiate further conjugation.

### 3.2. Bacteria Colony Count

**Figure 5:**
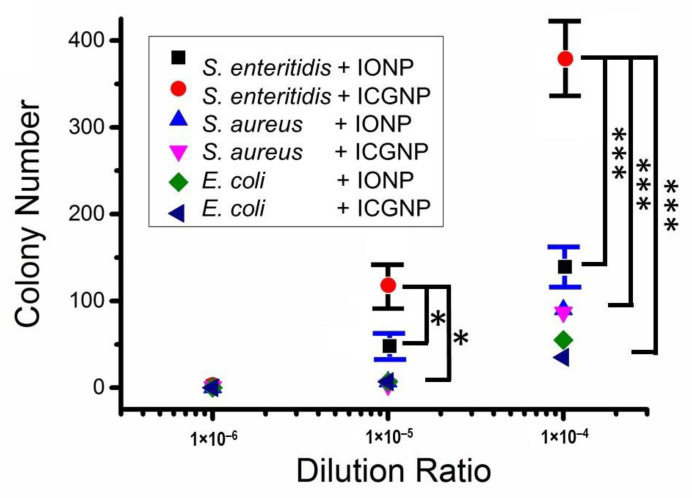
Incubation of different bacteria, dilution ratios, and NPs after 24 h. IONPs indicate IONPs conjugated with anti-Salmonella antibodies and ICGNPs indicate ICGNPs conjugated with anti-Salmonella antibodies. *, *p* < 0.05, ***, *p* < 0.001.

First, the colony growth of *S. enteritidis*, *S. aureus*, and *E. coli* without nanoparticles was monitored on the agar plate. 100 μL of conjugated ICGNPs were mixed with 0.9 mL each of different dilutions from 10^−6^ to 10^−1^. As shown in Figure 5, the different dilutions produce different colony numbers. The colony number obtained for *S. enteritidis* was much higher than that for *S. aureus* and even higher than for *E. coli*. Statistically relevant differences are observed between *S. enteritidis +* ICGNPs and *S. aureus* + ICGNPs and *E. coli* + ICGNPs *at dilution* 10^−5^ (*p* < 0.05) and 10^−4^ (*p* < 0.001). Colony numbers for *S. enteritidis* + ICGNPs were higher than *S. enteritidis* + IONPs. There is a statistically relevant difference between ICGNPs and IONPs on *Salmonella at dilution* 10^−5^ (*p* < 0.05) and 10^−4^ (*p* < 0.001).

The percentage of colonies indicates the efficiencies of ICGNPs to gather bacteria. The number of colony growth without NPs was recorded. Then the number of colony growth with IONPs and ICGNPs was compared to that without nanoparticles. The *S. enteritidis* number was 1 to 10^5^ following dilution 10^−6^ to 10^−1^, respectively. The colony number for ICGNPs is higher than other groups at the same dilution. The efficiencies of ICGNPs to gather bacteria for *S. enteritidis* were 20%, 32%, and 40% at concentrations of 10 to 1000 CFU/mL, respectively. There were statistically relevant differences between the ICGNPs to IONPs and other groups at 1000 CFU/mL (*p* < 0.001) at 100 CFU/mL (*p* < 0.05). There are statistically relevant differences between the IONPs and other groups without nanoparticles at 1000 CFU/mL (*p* < 0.05).

### 3.3. Fluorescent Detection

**Figure 6:**
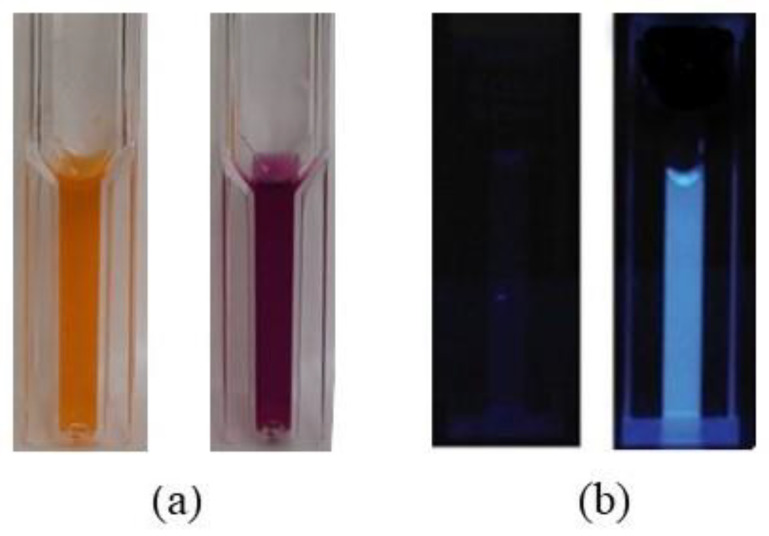
Florescent test of *Salmonella* interaction with conjugated ICGNPs (**a**) With white light. (**b**) With UV light.

The detection of *Salmonella* at 100 CFU/mL concentration by ICGNPs or IONPs was performed in two different cuvettes. After enrichment according to the protocol of Section 2.6, 0.9 mL of buffered 4-MUCAP substrate solution was injected into the cuvette. The magnets were applied to attract ICGNPs and IONPs with *Salmonella* and 4-MUCAP. The solution was decanted, and the particles were resuspended with ultrapure water in the cuvette. The cuvette was imaged under white light, as shown in Figure 6a. IONPs were orange in color with *Salmonella*, while ICGNPs were purple. Figure 6b illustrates that, with the UV light in a fluorescent test, the IONPs sample was dark or did not show intensive color, while, in contrast, the ICGNPs gave bright blue fluorescence in the presence of *Salmonella*. No bright blue fluorescence was discernable when the concentration of bacteria was lower than 100 CFU/mL, indicating that ICGNPs can detect *Salmonella* at 100 CFU/mL. When magnetic multiplex conjugated with 20 µg/mL of anti-Salmonella antibody, the efficiency of ICGNPs gathering *S. enteritidis* was 32% at 100 CFU/mL concentration. The limit of detection was 32 *Salmonella* per mL. On the other hand, the efficiency of IONPs gathering *S. enteritidis* was 15% at 1000 CFU/mL concentration. The limit of detection was 150 *Salmonella* per mL.

### 3.4. Sensitivity Test

In Table 1, the statistical *p* value of Fisher’s exact test for IONPs and ICGNPs were 0.0374 and 0.0022, and all results were significant at *p < 0.05*. The statistical predictions given explained that both IONPs and ICGNPs methods are successful. However, the ICGNPs were predicted to be relatively better. For example, there were five positives out of six *Salmonella* contaminated samples with the ICGNP method. On the other hand, there were four positives out of six *Salmonella* contaminated samples with the IONP method.

In Table 2, it is shown that the ICGNPs have higher sensitivity, specificity, Positive Predictive Value (PPV) and Negative Predictive Value (NPV) than IONPs. A 95% confidence interval (CI) of the mean is a range with an upper and lower number calculated from a sample [30]. The standard normal distribution, 95% of the CI range, was narrower for ICGNPs than IONPs. So, in monitoring the *Salmonella*, ICGNPs could be a better choice than IONPs. The sensitivity, specificity, Positive Predictive Value (PPV) and Negative Predictive Value (NPV) were 83.3%, 92.9%, 83.3% and 92.9%, respectively. The four parameters for ICGNPs were higher than those for IONPs. This means that the ICGNPs method is better at both confirming the presence (PPV) and the absence (NPV) of *Salmonella*.

## 4. Discussion

Iron oxide nanoparticles (IONPs) and iron core gold nanoparticles (ICGNPs) were synthesized and characterized during this study, by the reduction and seed growth method, respectively (Figure 1). VSM results confirmed the magnetic property of synthesis of nanomaterials (Figure 2a,b). The XRD characteristic Fe crystalline pattern corresponding to peaks at 2θ = ~30°, ~35°, ~43°, ~53°, ~57°, ~63°, ~74°, which are characteristic iron crystalline pattern corresponding to Fe (220), (311), (400), (422), (511), (440), (533) in IONPs from both fcc and bcc [25,26,27,28]. XRD pattern of ICGNPs exhibits peaks at 2θ = ~38°, ~45°, ~65 °, ~78° and ~82°. The peaks at 2θ = ~38°, ~45°, ~65 °and ~78° are characteristic of gold crystalline diffraction, corresponding to Au (111), (200), (220) and (311). The overlap of their diffraction peaks at 45°, 65° and 82° are corresponding Fe (110), (220) and (211) [29]. The TEM images showed that the IONPs are spherical with a diameter of 10 nm while the ICGNPs were flower/spike shaped with a hydrodynamic diameter of 30 nm (Figure 3b, Figure 4a and Figure S1b). The ICGNPs are heterogeneous nanoparticles, and the role of citrate in the growth of the heterogeneous nanoparticle surface is illustrated in the current study. Gold atoms are deposited on the IONP surface through the chemistry of the carboxylate group [31]. In general, the citrate ions of the seed surface are tightly bound, limiting the growth rates [32]. However, in the places where citrate ions are less tightly bound or unbound, the growth rates are relatively fast. As a result, the varied growth rates at different curvatures and planes of the precursor particles lead to the appearance of the spikes on the surface of IONPs particles [33]. The XRD confirmed ICGNP with the core iron and full layer of gold shell. The core diameter of ICGNP is 22 nm. There are 15 spikes on the surface of ICGNP. The total surface area of ICGNP was calculated to be 2059 nm^2^ per particle, compared to 334 nm^2^ per IONP, which provides a larger surface area to react with antibodies (Figure S2, Table S1).

In the mechanism proposed by Zou and coauthors, it is expected that citrate ions should be loosely distributed on the surface of gold particles, due to the repulsion of the negatively charged citrate ions [33]. IONPs and ICGNPs are magnetic and unstable and lack control over the dispersion of the particles in a solvent. Therefore, a solution of citric acid (coordinates with a carboxyl group) is used to stabilize the NPs [26]. Carboxylic acid groups adsorb on iron and gold surfaces under acidic conditions [26,31]. The current study shows citrate on the surface of IONP–COOH and ICGNP–COOH after citrate acid treatment for 3 h at temperature 80 °C (Figure 4f). The average particle hydrodynamic size of ICGNP was increased to 100, 185, 300 and 1500 nm after conjugation with -COOH, (EDC and NHS), and *(Salmonella* and its antibody) respectively (Figure 4). Conjugation of the nanoparticles with antibodies plays an important role in biosensing and biosensors, to develop new assays and diagnostic devices. EDC and NHS allow the coupling of proteins and increase the stability by conjugating with -COOH group on nanoparticles’ surfaces. The conjugation reaction was started when the solution pH was equal to 7. The reaction stopped when the solution pH was equal to 9. The anti-Salmonella antibody conjugation is performed by following the Bradford method.

This method involves the use of the fluorescence dye, 4-MUCAP, which acts as an indicator due to its high fluorescence intensity at pH 6.8 [22]. The response can be photographed under UV light from a camera exposure chamber. The dye 4-MUCAP ester is efficient because it is a fluorogenic substrate to C8 esterase which is very compatible with *Salmonella*, and it also functions best at pH 6.8. When ICGNPs were conjugated with 20 μg/mL of anti-Salmonella antibody, the efficiency of gathering *Salmonella* was 32% for 100 CFUs/mL (Figure 5 and Figure 6). Using ICGNPs to enrich the bacteria which then glows under UV conditions shows the presence or absence of bacteria (Figure 6b).

Standard bacterial cultures on agar plates are accurate but can take up to 7 days to identify the contaminating bacteria [34,35]. The diagnosis of infection versus colonization with these bacteria is time-consuming. The agar plate incubation is slow and expensive, but useful to confirm the presence and the absence of bacteria and their characterization. It usually takes 2 days to declare positive or negative results after culturing on agar plates. Current ICGNP method takes 20 min. Notably, the ICGNP method is much simpler and faster. The specificity and sensitivity of the IONP and ICGNP methods have been evaluated. The ICGNP method has 83.3% sensitivity and a 92.9% specificity for the presence of *Salmonella* (Table 2). The negative predictive value (NPV) is the percentage of patients with a negative test who do not have the presence of *Salmonella*. NPV tells us how many of the test negatives true negatives are; and if this number is 90%, then it suggests that agar plate incubation is doing as well as the gold standard techniques. Moreover, the ICGNP method (100 µL per test) represents a dramatic reduction in the cost of generating results, and the ICGNP method delivers precise results of bacterial detection after 10 min of bacterial enrichment and 10 min of fluorescent dyes enrichment with magnet.

One advantage is the rapid culture, the other is the visible detection. It is perfectly suited for analyses in laboratories, public places, or farms/livestock infrastructures. IONPs have the benefits of being easily synthesized, high bioavailability, and super paramagnetism, and are widely used in nanotechnology. IONPs are also relatively easy and cheap to work with. The toxicity of gold has been confirmed as low on animal cells and fungi while iron has been found toxic to marine organisms and mammals [36,37,38]. ICGNPs can conjugate with 4-MUCAP fluorescent dyes and shows visual results of bacterial presence or absence. Therefore, ICGNPs are the better choice for the design and development of LFIA. In summary, ICGNPs have better properties than IONPs to monitor *Salmonella*. Gessner and coauthors have successfully decorated magnetic nano-particles with gold nano particles [20]. In contrast, in the current study, the XDR shows an iron core- gold shell structure, indicating that a gold layer fully covers the surface of the IONPs. The advantage of this method is that ICGNPs can be easy conjugated over their large surface area and retain their magnetic for separation in a magnetic field.

The new technologies combined with test strips is used in the field of food safety testing, such as aptamers, surface-enhanced Raman spectroscopy, quantum dots, electrochemical test strip detection technology, biosensor test strip detection [39]. The commonly used methods with relatively high sensitivity and shorter time for the monitoring of *Salmonella* and other bacterial contaminated samples are PCR and LFIA, details relating to the performance of which are shown in Table 3.

PCR has high sensitivity (10–1000 CFU/mL), which is the main reason PCR is considered a reliable method for the detection of microbes [5,6]. Another advantage is that the PCR can be performed on the raw food samples directly without any sample preparation. However, it needs complex and extensive procedures with well-trained staff, and it takes time to complete a reaction which limits its onsite real-time usage to monitor bacterial contaminants [5]. LFIA on the other hand takes less time and needs a few steps. Therefore, LFIA can be performed by everyone conveniently at any place with rapid results [8]. However, drawback is that LFIA cannot be performed on the raw food sample directly, and the food sample must be placed in the solution first [7,8,43,44]. Additionally, the sensitivity of LFIA is lower than PCR (100–50,000 CFU/mL), which means results from LFIA may not be as accurate and reliable as that of a PCR. In summary, both PCR and LFIA have advantages and disadvantages and are suitable in different circumstances. Phenoxy-dioxetane luminescence probes has been developed for the limits of detection is 10 *Salmonella*. However, the incubation time is 6 h with multiple reaction process [45]. The advantage of this method is the image resolution and monitoring sensitivity with magnetic bacterial enrichment and the fluorescence dye, 4-MUCAP with in 20 min. The advanced software system can be used to analyze the photograph captured by using the smartphone to quantify the concentration of bacterial contaminants in the water samples [46,47]. Recently, the AuNPs/Au electrode and *Salmonella* phage L66 (phage L66) has been employed to analysis the electron transfer ability for senor of *Salmonella* [42]. The limit of detection (LOD) of the established method is 13 CFU/mL which is lower detection line than literatures [42]. The current work shows that the limit of detection of ICGNPs conjugated with Salmonella antibody is 32 *Salmonella enteritidis* per mL with specificity of 92.9%. In future, the define method with different antibodies can be employed for different pathogen detections.

**Figure 7:**
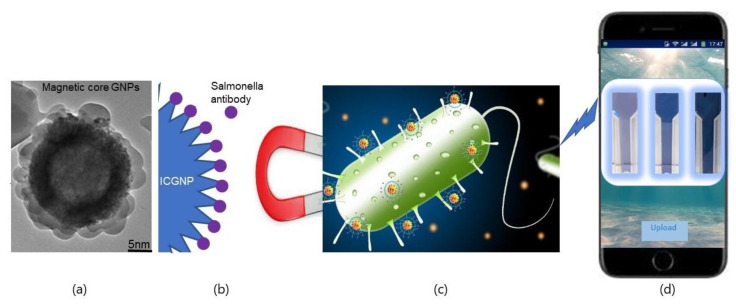
The procedure of monitoring *Salmonella* by a rapid magnetic fluorescent assay (**a**) TEM image of ICGNP; (**b**) ICGNP-COOH can be conjugated with antibody; (**c**) Magnetic enrichment for *Salmonella*; (**d**) Quantification of fluorescent intensity.

Figure 7 graphically illustrates the design and the functional concept of a rapid magnetic fluorescent assay to monitor *Salmonella* contaminated samples. It also explains the possibilities of the colorimetric analysis of the images of the samples captured after the completion of the reaction and their analysis. Figure 7a shows the TEM image of the flower-shaped ICGNP. This structure has many advantages as the gold coating does not compromise the magnetic effect that the iron provides for the magnetic separation while being enhanced with the qualities of the gold. Figure 7b shows the synthesis and addition of the carboxylate chain are necessary and it is what citric acid bind to, to increase the stability of ICGNPs. Moreover, the anti-Salmonella antibody with ICGNPs attracts *Salmonella*. Figure 7c represents how ICGNPs will wrap around *Salmonella* and attract *Salmonella* and they will be attracted to the external magnetic field. Figure 7d visualizes this research’s future, indicating that the result in an image can then be uploaded and analyzed to obtain a quantitative result.

## 5. Conclusions

Herein, we reported the synthesis of iron oxide nanoparticles (IONPs) and iron core gold nanoparticles (ICGNPs) by the reduction and seed growth method. XRD results confirmed gold layer is existing on the iron seed. The TEM images showed that the IONPs are spherical with a diameter of 10 nm while the ICGNPs were flower/spike shaped with a diameter of 30 nm. The ratio of surface area is 0.76 (IONPs/ICGNPs). The average particle size of ICGNP was increased to 100, 185, 300, and 1500 nm after conjugation with -COOH, (EDC and NHS), and (*Salmonella* and its antibody) respectively. The presence of a fluorescent dye helps to detect *Salmonella* under both white light (purple) and UV light (blue). In the bacteria incubation assay, the colony number for *Salmonella enteritidis* was much higher than *Staphylococcus aureus* and *Escherichia coli* when it was enriched with ICGNPs than IONPs. The limit of detection of ICGNPs conjugated with Salmonella antibody is 32 *Salmonella* per mL. The test had an 83.3% sensitivity and a 92.9% specificity value for the presence of *Salmonella*. The higher surface areas cause ICGNPs to have higher efficiency than the IONPs. The *Salmonella* contaminated samples can be monitored and reported rapidly by a rapid magnetic fluorescent assay. Users can get the qualitative result of *Salmonella* with the naked eye. Researchers can also obtain quantitative results by taking photographs of the strip on a smartphone and using software to analyze them.

## Figures and Tables

**Table 1:** Two-tail Fisher’s test for IONPs and ICGNPs.

*Salmonella*	IONP		ICGNP	
	With	Without	With	Without
Positive	4	2	5	1
Negative	2	12	1	13

**Table 2:** Comparison between IONPs and ICGNPs methods on double-blind *Salmonella* samples.

	IONPs		ICGNPs	
Statistic	Value	95% CI	Value	95% CI
Sensitivity	66.7%	22.3–95.7%	83.3%	35.9–99.3%
Specificity	83.3%	57.2–98.2%	92.9%	66.1–99.8%
Positive Predictive Value (PPV)	66.7%	33.0–89.1%	83.3%	42.2–97.2%
Negative Predictive Value (NPV)	83.3%	65.5–95.0%	92.9%	68.4–98.7%

**Table 3:** Comparison of different methods in literature for *Salmonella* detection.

Method	Limit (CFU/mL)	Sample	Samples	Ref.
PCR	1	24	Eggs	[38]
PCR	4	60	beef	[40]
PCR	10	96	Milk	[4]
PCR	1000	420	Seafood	[5]
LFIA	100	12	Bacteria solution	[7]
LFIA	1000	50	Bacteria solution	[40]
LFIA	10,000	72	Bacteria solution	[41]
LFIA	50,000	24	Bacteria solution	[6]
Electrochemical	13	3	Bacteria solution	[42]

## Data Availability

The data presented in this study are available on request from the corresponding author.

## Supplementary Materials

The following supporting information can be downloaded at: https://www.mdpi.com/article/10.3390/nano12213917/s1, Figure S1: TEM images with large views and scale bars; Figure S2: Surface area calculation for IONPs and ICGNPs; Table S1: The diameter of the core of ICGNPs, angle of the spike, and length of the spike of ICGNPs measurement with image J.
