# Supplementary material for: Design and Development of Magnetic Iron Core Gold Nanoparticle-Based Fluorescent Multiplex Assay to Detect Salmonella
Source: Nanomaterials (Basel). 2022 Nov 7;12(21):3917. doi: 10.3390/nano12213917 (PMC9655581; doi:10.3390/nano12213917)
Supplement: Supplementary file 1 [file nanomaterials-12-03917-s001.zip › nanomaterials-1991273-supplementary.pdf]

nanomaterials

MDPI

Supplemental material

Article

Design and Development of Magnetic Iron Core Gold Nanoparticle-Based Fluorescent Multiplex Assay to Detect *Salmonella*

Xinyi Zhao [1,2], Gwendoline Smith [1], Bilal Javed [1,2], Garret Dee [3], Yurii K. Gun'ko [3], James Curtin [4], Hugh J. Byrne [2], Christine O'Connor [1] and Furong Tian [1,*]

1 School of Food Science & Environmental Health, Technological University Dublin, Grangegorman, D07 H6K8, Dublin, Ireland
2 FOCAS Research Institute, Technological University Dublin, Camden Row, D08 CKP1, Dublin, Ireland
3 AMBER, Trinity College Dublin, D02 PN40, Dublin, Ireland
4 Faculty of Engineering and Built Environment, Technological University Dublin, Bolton Street, D01 K822 Dublin, Ireland
* Correspondence: furong.tian@tudublin.ie

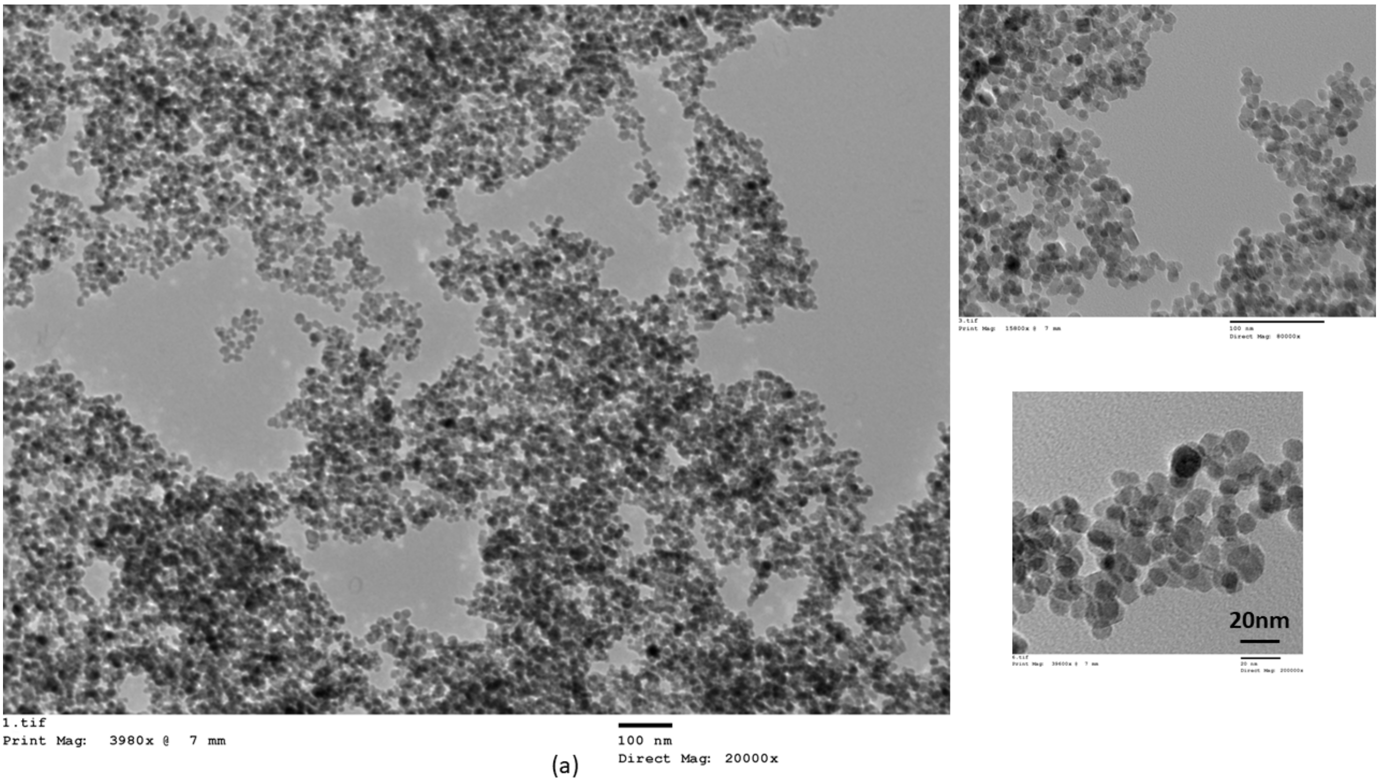

(a)

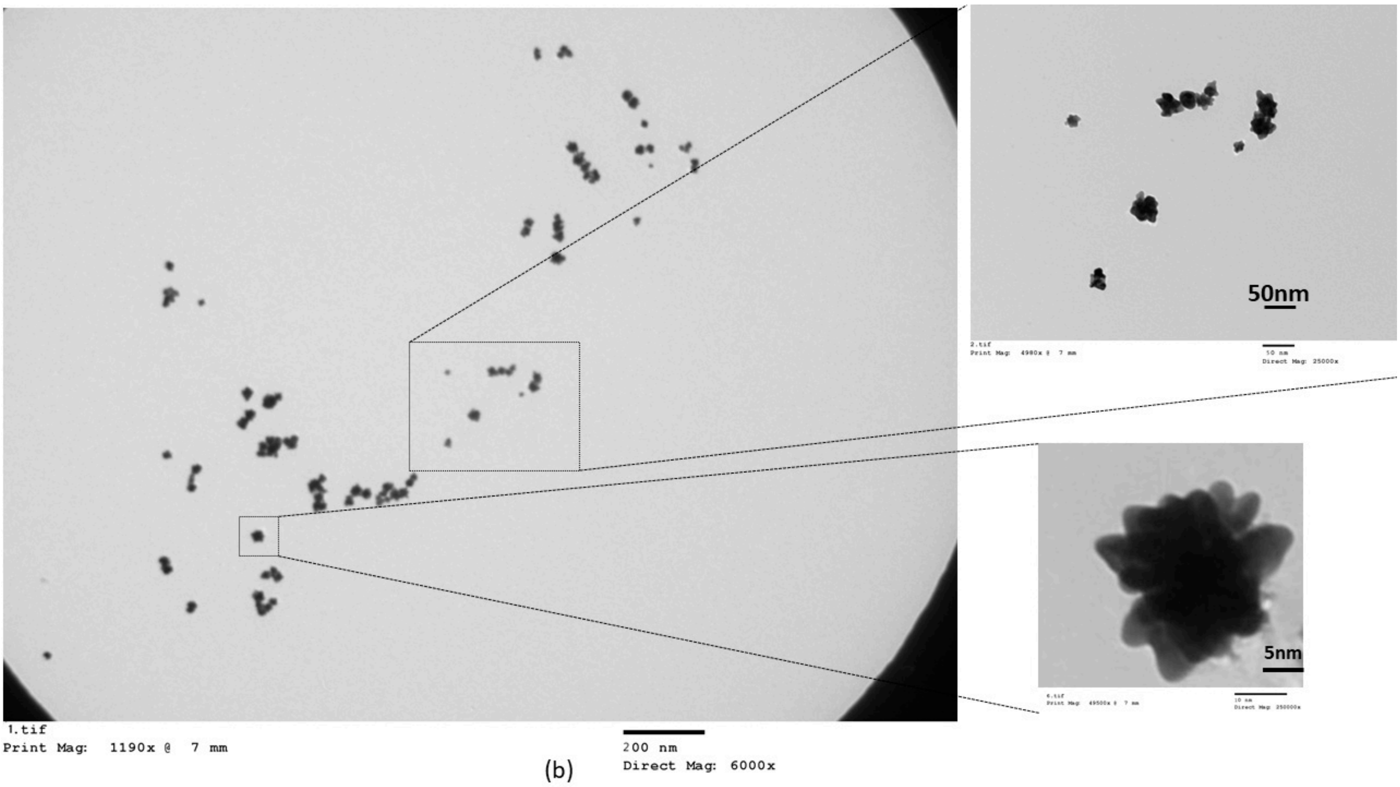

Figure S1. TEM images with large views and scale bars. (a) IONPs at different magnifications. (b) ICGNPs. The part of the image in the regular shape was magnified at top of the right image. The part of the image in the square shape was magnified at the bottom right image.

In order to study nanoparticle geometry, Image J was employed. The threshold analysis of images was set to highlight particles in black and white. The particle area is obtained by the measurement of the area occupied by black. The particle area, length, and angle of the spike were determined and collated (S table 1). The diameter of the core of ICGNPs was 22± 1.5 nm. The average angle was 41 ± 1.1 degrees. The average length spike was 7.1± 0.2.

Table S1. The diameter of the core of ICGNPs, angle of the spike, and length of the spike of ICGNPs measurement with image J.

Diameter of Core of ICGNPs	Angle degree	Length nm
18.684	39.01	7.061
21.324	39.538	7.031
22.124	41.226	6.848
23.864	39.289	7.296
21.944	39.199	6.718
21.867	40.435	7.344
22.812	42.989	6.86
22.329	39.806	7.009
21.128	40.843	7.066
23.488	39.26	6.941
21.987	40.141	6.86
19.944	42.784	7.119

19.913	38.009	7.098
23.002	39.366	7.611
23.91	40.435	7.072
19.184	40.62	6.68
18.934	39.932	6.961
22.155	40.46	6.959
23.124	40.854	7.124
22.627	41.891	7.013
23.152	40.13	7.143
22.179	40.699	6.982
23.118	41.821	6.791
23.142	40.234	7.265
22.767	41.332	7.143
22.441	40.457	6.986

Based on idealized geometric structures, the surface area of ICGNP with core shaped spike are illustrated in Figure S2. The IONPs are shown as red spherical nanoparticles as seeds of ICGNPs. A perfect spherical geometry is assumed for the core of ICGNPs.

The spike on the surface of the core of ICGNP is calculated as a cone, having a. Lateral surface and a Base area, the latter of which is not included in the calculation of the NP surface area.

Equation 1 describes the Total surface area of an ICGNP
$A_T = A_C + NA_L - NA_B$. (Equation 1)
A_T:Total surface area of ICGNP
A_C: Surface area of the core of ICGNP
A_L: Lateral surface area of a core
A_B: Base area of a core
N: Number of corns on the surface of the core of ICGNP

Surface area of core of ICGNP
A_C: $4\pi(D/2)^2$
Lateral surface area of a cone:
$A_L = \pi rl$
Base surface area of a cone (a circle):
$A_B = \pi r^2$
N=15
Total surface area of a ICGNP:
$A_T = 4\pi(D/2)^2 + 15x\ \pi rl - 15\ x\ \pi r^2$ (Equation 2)
D: Diameter of core of ICGNP
l: Slant height of a cone:
r: Radio of a cone

The surface area of an IONPs is 314 nm^2 per particle. The surface area of spike ICGNPs were illustrated in Figure S2. The gold salt was deposited on the surface of IONP to form ICGNPs. The average diameter of the core of IONPs was 22 nm. The surface area of the core of ICGNPs was 1520 nm^2. If a perfect cone geometry is assumed for a spike of ICGNPs, the surface area was calculated as the number of cones times each core. Each spike was considered a cone. The number of spikes can be counted by Image J (Figure S1 and Table S1). The base of the cone had average diameter of the 5 nm and area 19 nm^2. 15 cones were counted with Image J. The length of the vertices was averaged at 7 nm. The length of the spike was seen as a slant in the cone (Figure 4). The lateral surface area of a

cone (A_L) was 45 nm^2. The lateral surface area of 15 cones was 824 nm^2. The 15 base surface area of cones was 285 nm^2. The total surface area of an ICGNP was 2,059 nm^2 per particle (Equation 2). The total number of IONPs was 5 times of ICGNPs. However, the surface area was lower than the ICGNPs.

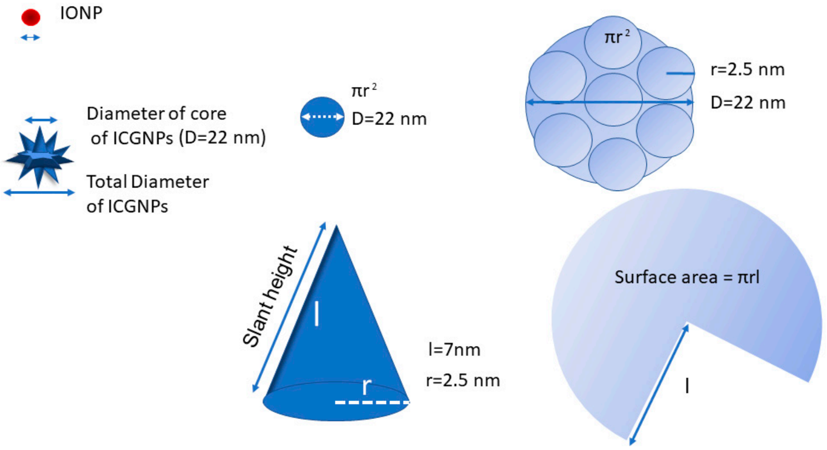

Figure S2. Surface area calculation for IONPs and ICGNPs.
